# Intussusception Caused by Percutaneous Endoscopic Gastrostomy With Jejunal Extension in Patients With Severe Motor and Intellectual Disabilities

**DOI:** 10.1097/PG9.0000000000000088

**Published:** 2021-06-15

**Authors:** Shin-ichiro Hagiwara, Takatoshi Maeyama, Hitoshi Honma, Hideki Soh, Noriaki Usui, Yuri Etani

**Affiliations:** From the * Department of Gastroenterology, Nutrition and Endocrinology, Osaka Women’s and Children’s Hospital, Izumi, Osaka, Japan; † Department of Pediatric Surgery, Osaka Women’s and Children’s Hospital, Izumi, Osaka, Japan; ‡ Department of Pediatric Surgery, Kawasaki Medical School, Kurashiki, Okayama, Japan.

**Keywords:** enteral nutrition, intussusception, jejunal tube feeding, long-term use

## Abstract

The risk of intussusception related to percutaneous endoscopic gastrostomy with jejunal extension (PEG-J) in patients with severe motor and intellectual disabilities (SMID) remains unknown. In a cross-sectional study, a review of 26 patients (mean age, 11.6 ± 6.4 years) with SMID who underwent PEG-J was performed. During the follow-up period, 6 of 26 (23%) patients developed intussusception. The median period from PEG-J to the onset of intussusception was 364 (range, 8–1344) days. No significant difference was observed in the Cobb angle between the intussusception and nonintussusception groups; however, body mass index at the time of PEG-J was significantly lower in the intussusception group. Intussusception related to PEG-J occurs relatively frequently in patients, and it is possibly attributable to factors such as deformity caused by undernutrition and weight loss. If enteral nutrition via PEG-J has been established, earlier enterostomy can be recommended because of the high risk of intussusception in patients with SMID.

**What Is Known**

- Percutaneous endoscopic gastrostomy with jejunal extension (PEG-J) is an alternative technique for patients with severe motor and intellectual disabilities (SMID). The incidence of intussusception in children with SMID is unknown.

**What Is New**

- About one-fifth of patients with SMID developed intussusception. Long-term PEG-J insertion is not recommended in patients with SMID because of increased risk of intussusception development by PEG-J.

## INTRODUCTION

Pediatric patients with severe motor and intellectual disabilities (SMID) occasionally experience oral feeding difficulties. Thus, feeding methods such as tube feeding and percutaneous endoscopic gastrostomy (PEG) are chosen. As patients grow, various factors such as repeated aspiration because of gastroesophageal reflux disease (GERD), decreased gastric motility, and intestinal obstruction caused by scoliosis (eg, superior mesenteric artery syndrome) may lead to difficulties with tube and PEG feeding (1,2). PEG with jejunal extension (PEG-J) is an alternative technique for patients who cannot tolerate gastric feeding, including those with gastroparesis, severe GERD, a history of repeated aspiration, gastric resection, or gastric outlet obstruction (3). PEG-J is also a useful nutritional route for children with SMID (4). Although PEG-J is relatively safe, complications have been reported^5^–^8^. Serious complications include intussusception, but the incidence of intussusception in children with SMID is unknown. We present our experience at a tertiary children’s hospital to ascertain the incidence and risk factors of intussusception caused by PEG-J.

## METHODS

In a cross-sectional study, the outcomes of 26 patients (12 males and 14 females) who underwent PEG-J following unsuccessful gastric feeding at Osaka Women’s and Children’s Hospital between April 2012 and March 2019 were retrospectively reviewed. All patients fulfilled the criteria of classes 1–4 of Ohshima’s classification (9). Ohshima classified the level of each motor and intellectual disability by combining them into 25 different divisions. Among 25 divisions, class 1 is used to describe people who are bedridden and have an IQ lower than 20; class 2 describes those who are able to sit and have an IQ lower than 20; class 3 describes those who are able to sit have an IQ between 20 and 35; and class 4 describes those who are bedridden and have an IQ between 20 and 35 (9). Data regarding baseline characteristics such as age, gender, comorbidities, Cobb’s angle, and body mass index (BMI) at the time of PEG-J and airway management were compared between patients with and without intussusception. The severity of scoliosis was defined by the Cobb angle as mild (0°–60°) or severe (>60°). A 16-Fr all-silicone jejunal tube (effective length, 400 or 600 mm) with gastric decompression function (Cliny PEG-J Catheter, Create Medic Co., Ltd, Yokohama, Japan) was used, and all procedures were performed in an interventional radiology suite with the use of fluoroscopy. The study protocol was approved by the Ethics Committee of Osaka Women’s and Children’s Hospital (Registration number 2019-1242). Statistical analysis was performed using STATA/SE 13.1 for Mac (StataCorp, College Station, TX). Continuous variables are presented as the mean ± SD or median (range) as appropriate. Categorical variables are expressed as numbers and percentages. Pearson’s chi-square test was used to compare categorical variables between the groups, and Mann–Whitney’s U test was used to compare continuous variables between the groups. Kaplan–Meier survival analysis was performed to estimate the risk of intussusception. Significance was indicated by *P* < 0.05. Institutional review board approved was obtained (2020-1242).

## RESULTS

**FIGURE 1.**
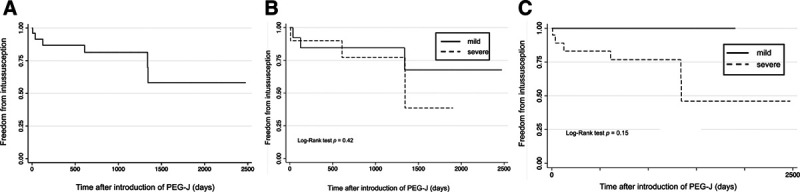
Kaplan–Meier survival curves displaying the estimated intussusception-free probability after percutaneous endoscopic gastrostomy with jejunal extension (PEG-J)A) A patient who underwent PEG-J. B) The severity of scoliosis was indicated by the Cobb angle as mild (solid line, 0°–60°) or severe (dashed line, >60°). C) The severity of undernutrition was indicated as mild (solid line, ≥15) or severe (dashed line, <15) on the basis of the body mass index.

Patient characteristics are listed in Table 1. The overall success rate of PEG-J in our cohort was 100%. The mean patient age was 11.6 ± 6.4 years. The median period between PEG and PEG-J was 1795.4 (range, 57–5653) days. During the follow-up period, 6 of 26 (23%) patients developed intussusception (3 cases; jejuno-jejunal type, 3 cases; duodenal-jejunal type). Of these patients with intussusception, 4 cases were diagnosed using abdominal ultrasound and 2 using abdominal ultrasound as well as computed tomography. No features of arterial or venous compromise were observed. Indwelling PEG-J catheter led to intussusception by acting as a lead point in all cases. All patients were treated by manual PEG-J removal under echo guidance, and no subsequent accident was reported in all cases. The initial symptoms of intussusception were vomiting (2 cases), tachycardia (2 cases), and bile reflux (1 case), and 1 patient was asymptomatic. Intussusception was accidentally identified when using abdominal ultrasound for a follow-up of gallstones in the asymptomatic patient. The median period from PEG-J to the onset of intussusception was 364 (range, 8–1344) days. Of the 6 patients who developed intussusception, four patients underwent enterostomy following intussusception. No significant difference was observed in the Cobb angle between the intussusception and nonintussusception groups (67.8° versus 49.9°); however, BMI at the time of PEG-J was significantly lower in the intussusception group (10.3 versus 13.7, *P* = 0.004). In Kaplan–Meier survival analysis, almost half of the patients developed intussusception approximately 1500 days after PEG-J (Fig. 1A). The incidence of intussusception did not differ statistically according to the severity of scoliosis and undernutrition (Fig. 1B and C).

**TABLE 1.** Patient characteristics

Patients	Total (n = 26)	Intussusception (n = 6)	Nonintussusception (n = 20)	*P*
Sex (female), n (%)	14 (63)	3 (50)	11 (55)	
Asphyxia, n (%)	11 (42)	3 (50)	8 (40)	
Chromosomal disorder/syndrome, n (%)	9 (34)	1 (16)	8 (40)	
Tracheostomy, n (%)	18 (69)	5 (83)	13 (65)	
Laryngotracheal separation, n (%)	16 (61)	5 (83)	11 (55)	
Enterostomy, n (%)	8 (30)	4 (66)	4 (20)	
Continuation of PEG-J feeding. n (%)	9 (34)	1 (16)	8 (40)	
Age at PEG insertion, mean ± SD (range), years	6.3 ± 6.0 (0.4–21.5)	5.0 ± 6.2 (0.5–17.2)	6.7 ± 6.0 (0.4–21.5)	0.46
Age at PEG-J insertion, mean ± SD (range), years	11.6 ± 6.4 (2.6–25.7)	10.2 ± 5.8 (4.2–20.7)	12.0 ± 6.6 (2.6–25.7)	0.54
Time interval between PEG and PEG-J insertion, median (range), days	1795.4 (57–5653)	1297 (1223–2097)	1862 (57–5653)	0.74
Body weight at PEG-J insertion, mean ± SD (range), kg	17.9 ± 6.1 (8.9–29.3)	14.3 ± 5.3 (10.4–24.7)	19.1 ± 6.1 (8.9–29.3)	0.06
BMI at PEG-J insertion, mean ± SD (range)	12.9 ± 2.9 (8.9–20.2)	10.3 ± 1.2 (8.9–12.4)	13.7 ± 2.9 (10.3–20.2)	0.004
Duration of PEG-J use, median (range), days	827.9 ± 735.2 (3–2473)	367 (7–1344)	837 (3–2473)	0.41
Cobb angle at PEG-J insertion, mean ± SD (range), degree	54.0 ± 35.0 (0–128)	67.8 ± 38.7 (24–128)	49.9 ± 33.7 (0–116)	0.21
Body weight at onset of intussusception, mean ± SD (range), kg		15.5 ± 7.1 (10.3–29.4)		
BMI at onset of intussusception, mean ± SD (range)		11.3 ± 2.1 (8.9–15)		
Time interval between PEG-J insertion and intussusception, median (range), days		364 (8–1344)		

BMI = body mass index; PEG-J = percutaneous endoscopic gastrostomy with jejunal extension.

## DISCUSSION

Tube feeding or gastrostomy is commonly used if oral intake is insufficient or impossible for patients with SMID (10). However, some patients with SMID cannot tolerate gastrostomy because of GERD, decreased motility of stomach, and gastrointestinal obstruction (2,11). In such cases, enteral feeding with an ED tube or PEG-J is an alternative option. PEG-J was first reported by Ponsky et al in 1984 (12). PEG-J is useful for patients with chronic intestinal pseudoobstruction chronic pancreatitis and Parkinson’s disease (13–15). The efficacy of PEG-J has also been reported in pediatric patients (5–8,16). The most common complication of PEG-J is retrograde tube migration. Other complications include tube obstruction, tube fracture, perforation, peritoneal leakage, and small bowel intussusception (17). The incidence of intussusception ranges from 1.3% to 48.7% in pediatric populations (5,8,18). The mechanism of intussusception is unknown, but there are a number of possible causative factors (19,20). In adults, secondary intussusception is reportedly initiated at any pathologic lesion on the intestinal wall or an irritant within the lumen that alters normal peristaltic activity and serves as a lead point (21). We hypothesized that the tip of PEG-J plays the role of a “pathologic lesion” as the lead point for patients with SMID or provokes impaired intestinal peristalsis because of intestinal malposition due to severe scoliosis. Further studies are required to elucidate the pathogenesis of intussusception caused by PEG-J. Based on the present results, long-term PEG-J insertion is not recommended in patients with SMID. If the patients require prolonged PEG-J feeding, a transition to enterostomy should be considered.

## CONCLUSIONS

Physicians should consider the possibility of intussusception if patients undergoing PEG-J feeding exhibit unexplained vital changes. Additionally, low body weight and low BMI may predispose patients to intussusception, and enterostomy should be introduced as necessary.
